# Purification and characterization of RGA2, a Rho2 GTPase-activating protein from *Tinospora cordifolia*

**DOI:** 10.1007/s13205-016-0400-3

**Published:** 2016-03-01

**Authors:** Mohd. Amir, Mohammad Aasif Dar, Wahiduzzaman, Asimul Islam, Faizan Ahmad, Md. Imtaiyaz Hassan

**Affiliations:** Centre for Interdisciplinary Research in Basic Sciences, Jamia Millia Islamia, New Delhi, 110025 India

**Keywords:** *Tinospora cordifolia*, Chromatography, Protein purification, Rho GTPase activating protein, MALDI-TOF

## Abstract

Rho GTPases activating protein 2 (RGA2) is primarily involved in the modulation of numerous morphological events in eukaryotes. It protects plants by triggering the defense system which restricts the pathogen growth. This is the first report on the isolation, purification and characterization of RGA2 from the stems of *Tinospora cordifolia*, a medicinal plant. The RGA2 was purified using simple two-step process using DEAE-Hi-Trap FF and Superdex 200 chromatography columns, with a high yield. The purity of RGA2 was confirmed by SDS-PAGE and identified by MALDI-TOF/MS. The purified protein was further characterized for its secondary structural elements using the far-UV circular dichroism measurements. Our purification procedure is simple two-step process with high yield which can be further used to produce RGA2 for structural and functional studies.

**Electronic supplementary material:**

The online version of this article (doi:10.1007/s13205-016-0400-3) contains supplementary material, which is available to authorized users.

## Introduction

The Rho GTPases activating protein 2 (RGA2), belonging to the superfamily of small G proteins, is found in both higher and lower eukaryotic organisms. These proteins are involved in diverse biological processes such as regulation of actin and microtubule cytoskeleton, cell cycle, vesicle trafficking, cell polarity, and gene expression (Bustelo et al. 2007; Cansado et al. 2010). Enzymatically it has GTPase activity which hydrolyzes the GTP to GDP and inorganic phosphate (Bourne et al. 1991). The activity of RGA2 is positively regulated by GDP-GTP exchange which catalyzes the exchange of GDP for GTP to activate the switch. However, GTPase-activating proteins stimulate the intrinsic GTPase activity to inactivate the switch and GDP dissociation inhibitors which block spontaneous activation, are considered as a negative regulators (Soto et al. 2010). These regulators are important for the specificity of Rho functions (Villar-Tajadura et al. 2008). These regulators actually modify the signaling activity of the GTPase (de Bettignies et al. 2001).

The plant-specific Rop subfamily of Rho GTPases is very similar to the mammalian Cdc42 and Rac GTPases (de Bettignies et al. 2001). These Rho GTPases play significant roles in the regulation of peroxide-mediated cell death, calcium-dependent pollen tube growth, and other processes in plants (Roumanie et al. 2001; Wu et al. 2000). RGA2 is also found in higher plants where it acts as a small GTPase-associated defensome system for plant immunity (Liu et al. 2015). Furthermore, Rho GTPase activating proteins play important roles in the developmental process (Ye et al. 2014).

*Tinospora cordifolia* is a well-known Indian medicinal plant and its extract posses immunomodulatory and anticancer activities (Bala et al. 2015) especially against epithelial cancer cells (Maliyakkal et al. 2015). RGA2 has been reported to be purified and characterized from different sources. Yang and Watson (1993) reported that cloning, expression and characterization of rho, a ras-related small GTP-binding protein from the garden pea. Matsuda et al. (1996) have partially purified a membrane-associated GDP/GTP exchange proteins from the crude synaptic membrane fraction of rat brain using a combination of column chromatographies. RGA2 has been purified from *S. pombe* which was shown to regulates Rho2-Pck2 interaction and might participates in the regulation of the MAPK cell integrity pathway (Villar-Tajadura et al. 2008). Recently we have purified oligonucleotide binding (OB)-fold protein (Amir et al. 2015a) and Ras-related protein, Rab5a (Amir et al. 2015b) from the stem of *Tinospora cordifolia.*

In this article we are for the first time reporting the purification and characterization of RGA2, a Rho2 GTPase activating proteins from *Tinospora cordifolia*. We used a simple chromatographic procedure to get this protein in plenty with high purity. We also performed structural analysis to establish a structure–function relationship.

## Materials and methods

### Materials

*Tinospora cordifolia* plant was obtained from Ch. Devi Lal Rudraksh Vatika Herbal Nature Park, Bhudkalan, Yamunanagar, Haryana, India. The bark of green stem was removed by scalpel blade and homogenized in 50 mM Tris–HCl pH 8.0 with 500 mM NaCl. All reagents of highest purity grade were purchased from Sigma–Aldrich (St. Louis, MO, USA), Merck (Darmstadt, Germany) and GE Healthcare. Hi Trap DEAE FF and Superdex-200 columns were purchased from GE Healthcare, Uppsala, Sweden. Electrophoresis reagents were purchased from the Bio-Rad Laboratories (Richmond, CA, USA). Databases used are http://www.matrixscience.com/, http://www.uniprot.org., http://blast.ncbi.nlm.nih.gov/Blast.cgi and http://cbdm-01.zdv.uni-mainz.de/~andrade/k2d2//.

### Protein isolation

All purification steps were carried out at 4 °C. 200 g of green stem of *Tinospora cordifolia* without bark was crushed with 50 mM Tris–HCl pH 8.0 + 500 mM NaCl, and homogenized with blender in the ice-cold homogenization buffer (pH 8.0) containing 1 mM phenyl methyl sulphonyl fluoride (PMSF), 1 mM dithiothreitol (DTT), 1 mM ethylene diamine tetra acetate (EDTA), and 1 % polyvinyl pyrrolidone (PVP), 4 ml of buffer contained one gram of wet tissue. The homogenate was cleared by filtration through two layers of cheese cloth and left for overnight stirring at 4 °C. Solid ammonium sulfate was added to the homogenate to achieve 30 % saturation and kept for 12 h. Ammonium sulfate precipitate was removed after centrifugation at 12000*g* for 15 min. The supernatant thus obtained was further saturated to 60 % by ammonium sulfate and centrifuged at 12000*g* for 15 min. The supernatant obtained was further saturated to 90 % ammonium sulfate and centrifuged at 12000*g* for 15 min. The ammonium sulfate precipitate thus obtained was collected and dissolved in 50 mM Tris–HCl buffer, pH 8.0 and was extensively dialyzed against the same buffer.

### Ion-exchange chromatography

The dialyzed sample was loaded on Hi Trap DEAE FF (1 ml, 7 × 25 mm) column (GE Healthcare), pre-equilibrated with 50 mM Tris–HCl buffer, pH 8.0. Akta purifier (GE Healthcare) connected system to control the flow rate and fraction size of elution. The sample was injected into the column with 5 ml loop. The flow rate of 1 ml/min is maintained for both binding and elution. The column was washed with equilibration buffer, and the bound proteins were eluted with NaCl linear gradient (0–1 M NaCl w/v) in the same buffer. The first peak obtained at 0.10 M of NaCl was pooled, concentrated using Amicon Ultra 3 K device (Merck Darmstadt, Germany).

### Gel filtration chromatography

Concentrated protein sample (1 ml) was injected on to the Superdex 200 column connected to the Akta purifier (GE Healthcare, USA). The column was equilibrated with 50 mM Tris–HCl buffer, pH 8.0 at the rate of 0.5 ml/min. The elution profile was analyzed by unicorn manager (version 5.0) for the absorbance at 280 nm against elution volume (ml).

### Gel electrophoresis

Molecular mass of the protein was determined by SDS-PAGE as described by Laemmli (Laemmli 1970). The SDS-PAGE was performed in a slab gel assembly using 12 % (w/v) acrylamide and 0.02 % (w/v) bisacrylamide in the separating gel and 5 % (w/v) acrylamide and 0.16 % (w/v) bisacrylamide in the stacking gel. The gel buffer was 0.375 M Tris–HCl, pH 8.8. The electrode buffer was 25 mM Tris–HCl, pH 8.3 containing 0.192 M glycine. Gels were stained with coomassie brilliant blue G-250. Molecular mass standards (10–180 kDa) were used for the molecular mass determination.

### Mass spectrometry

The band of RGA2 was excised from the SDS-PAGE and subjected for identification to the matrix-assisted laser desorption/ionization time of flight (MALDI-TOF) (Kratos analytical, shimadzu group company, japan) equipped with a 337 nm pulsed UV laser, a 1.7 m length flight tube and a curved field reflectron. A detail of MALDI-TOF procedure was described elsewhere (Dar et al. 2014; Hassan et al. 2007, 2008b). The observed mass spectra, peak areas versus mass/electric charge (m/z) of mono-isotopic ions were calculated with MASCOT distiller software version1.1.2.0 (Matrix Science, London, UK).

### Circular dichroism measurements

Circular Dichroism (CD) spectra were measured in Jasco Spectropolarimeter (Model J-1500) equipped with peltier-type temperature controller (CDF-426S). The far-UV CD spectra (250–200 nm) were recorded at 25 ± 0.1 °C using a 1 mm path length cell, and the protein concentration used was 0.28 mg/ml. The samples were prepared in 50 mM Tris–HCl buffer at pH 8.0. The raw CD data were converted to protein concentration independent parameter, the mean residue ellipticity, (*θ*)_λ_ (deg cm^2^ dmol^−1^) using the relation, $\left[ \theta \right]_{\lambda } = M_{o} \theta_{\lambda } /10lc$ where *θ*_λ_ is the observed ellipticity in millidegrees, *M*_o_ is the mean residue weight of the protein, *c* is concentration in mg ml^−1^, and *l* is the path length of the cell in cm.

## Results and discussion

The root, stem, and leaves of *Tinospora cordifolia* are used in Ayurvedic medicine (Upadhyay et al. 2010). Phytochemicals present in the *Tinospora cordifolia* are used for the treatment of diabetes, high cholesterol, gout, allergic rhinitis, lymphoma and other cancers, upset stomach, rheumatoid arthritis, hepatitis, gonorrhea, syphilis, peptic ulcer disease, fever, and to boost the immune system (Saha and Ghosh 2012). Apart from several phytochemicals, this plant contains large number of proteins which has medicinal impact. Among these proteins, RGA2 is also one of the important protein, and having many clinical significance such as oocyte maturation, fertilization and early embryo development (Balakier et al. 2002). Structurally, RGA2 protein contains leucine-rich repeat (LRR) domains which are involved in diverse biological processes including signal transduction, cell adhesion and the innate immune response. Here, we report a rapid method for purification of RGA2 from *Tinospora cordifolia* for the first time followed by its structural characterization.

### Purification of RGA2

**Fig. 1:**
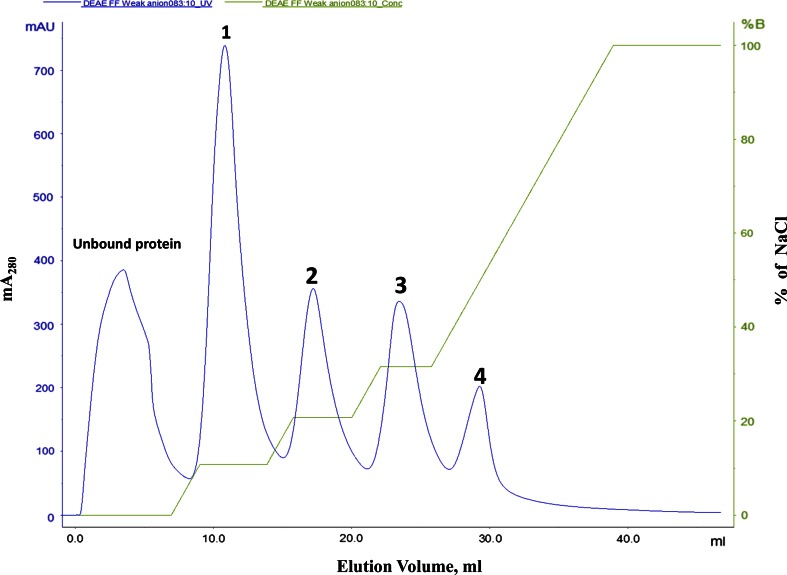
Elution profile [plot of mA280 (milli absorbance at 280 nm) versus elution volume (ml)] of weak anion exchanger Hi Trap DEAE FF. The second curve represents the gradient of NaCl (0–100 % of B) where buffer A is 10 mM Tris–HCl (pH 8.0) and B is 1.0 M NaCl in the same buffer

**Fig. 2:**
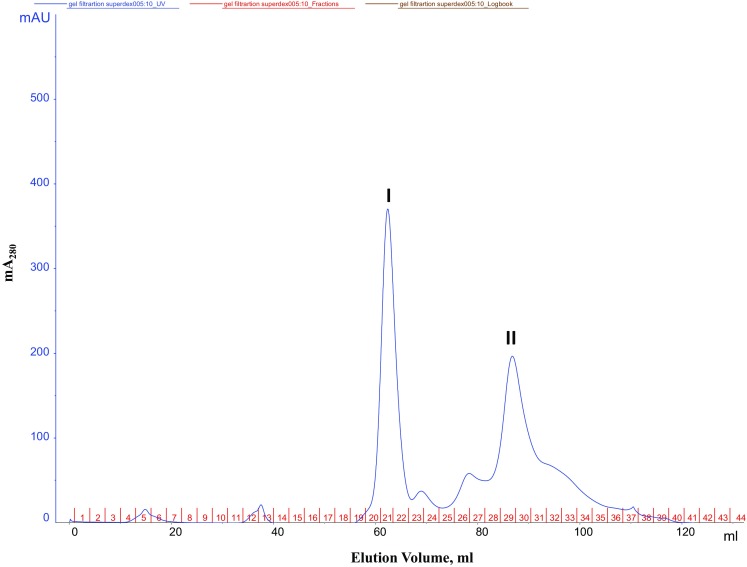
Elution profile of gel filtration superdex 200 column. The peaks were obtained as a function of X as elution volume in ml and *Y* axis is mA at 280 nm

**Fig. 3:**
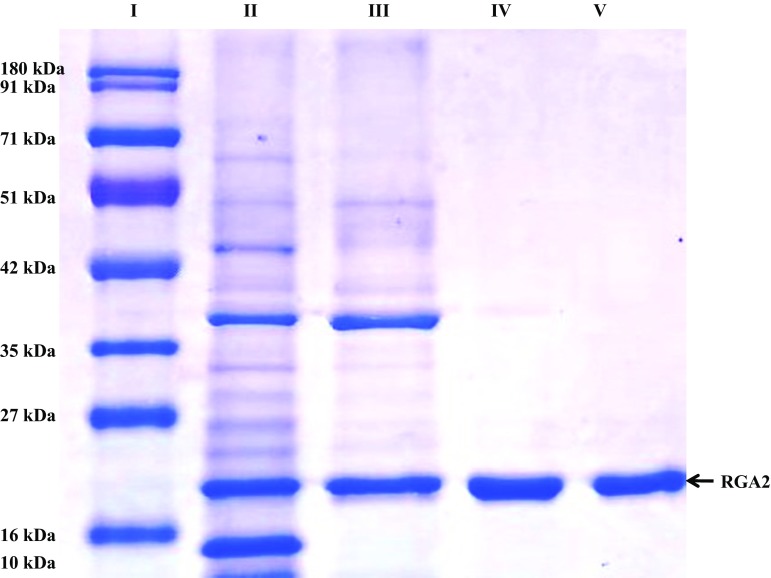
SDS-PAGE of peaks obtained during weak anion exchange and Gel filtration chromatography. Lane I is protein marker (10–180 kDa), Lane II is loading sample, Lane III is Pk1 of DEAE chromatogram and Lanes IV and V are PK II of gel filtration superdex 200 chromatogram

A summary of a typical purification procedure is given in Table 1. The dialyzed protein precipitated by 90 % ammonium sulfate was subjected to weak anion-exchange chromatography on Hi Trap DEAE FF. Bound proteins were eluted with a linear gradient of NaCl. We observed four peaks in the chromatogram of the weak anion-exchange elution (Fig. 1). The first peak, eluted at 0.10 M NaCl showing three prominent bands at 50, 35 and 20 kDa along with some low molecular mass impurities on SDS-PAGE. The concentrated pool of partially purified RGA2 protein from Hi Trap DEAE FF column was loaded on the Superdex 200 column, pre-equilibrated with 50 mM Tris buffer (pH 8.0). Proteins were eluted with the same buffer at a flow rate of 0.5 ml/min. Fractions of 2.0 ml were collected and monitored for absorbance at 280 nm (Fig. 2). Fractions showing high absorbance at 280 nm were pooled and concentrated for further analysis. The second peak showed a single band at 35 kDa (Fig. 3).

**Table 1:** Purification summary of RGA2

S.No.	Purification step	Volume (ml)	Protein (mg/ml)^b^	Total protein (mg)	Yield (100 %)
1	Crude extract^a^	880.0	1.8	1584	100
2	Ammonium sulfate (90 % cut)	50	1.6	80	5.05
3	DEAE column (pooled peak)	55	1.3	71	4.51
4	Superdex 200 column (pooled peak)	20	1	20	1.26

^a^From 200 g of wet weight of *T. cordifolia* stem

^b^Protein concentration determined by Lowry assay using BSA as a standard protein

### Protein identification

The single band shown in Fig. 3 was excised from the gel. This band after trypsinization was subjected to mass spectrometry. 13 fragments of different size were obtained after trypsinazation. Molecular mass and sequence of each fragments are shown in Table 2. These fragments were used for the identification of purified protein using mascot search program. A significant mascot score clearly indicates that the purified protein is RGA2 with sequence coverage of 35 %. Since, the sequence of RGA2 is not genbank therefore we considered RGA2 sequence of *Triticum aestivum* with GenBank: ACO55039.1 to identify the purified protein as RGA2. The purified RGA2 was freeze dried and stored for further uses.

**Table 2:** List of peptide fragments obtained after trypsinization

S. No.	Mass Mr.	Range	P sequence
1	973–5080	178–185	QLDTLDIR
2	1401–7517	1–12	RWIAEGYPGVVR
3	1415–7442	293–305	LGVMGINEKNDVK
4	1617–8991	199–212	LQKLQHLHAGFPTK
5	1645–8709	57–71	EIGISKSMEGNLVLR
6	2112–0687	202–219	LQHLHAGFPTKGNYLCTR
7	2118–9779	13–30	NKSTEEVAESYFMDLISR
8	2203–1136	111–128	SITVFGEWKPFFLSDKMR
9	2371–3032	178–198	QLDTLDIRGTSIVMLPQTIIK
10	2371–3032	178–198	QLDTLDIRGTSIVMLPQTIIK
11	2391–2659	265–286	SLHTIRGVHVAYGDAVIQEIGR
12	2821–3150	15–38	STEEVAESYFMDLISRSMLLPSQR
13	2821–3150	15–38	STEEVAESYFMDLISRSMLLPSQR

### Secondary structure measurement

Secondary structure measurement of protein using CD provided information that whether the purified protein is folded, and to checks its conformation or stability (Alam Khan et al. 2009, 2010; Greenfield 2006; Rehman et al. 2011; Singh et al. 2015). In addition, analysis of CD spectra helps to estimate the secondary structure composition of a protein (Anwer et al. 2014, 2015; Haque et al. 2015; Khan et al. 2016; Rahaman et al. 2015). Prediction of secondary structure for the CD data measured to 178 nm for α-helix: 0.97 for β-sheet: 0.75 for β-turn: 0.50 and for other structures: 0.89 (Manavalan and Johnson 1987). Structure analyses will also provide an insight into the function of protein (Hassan et al. 2008a, 2010, 2012, 2013). Hence, we performed CD measurements to estimate the secondary structure of purified RGA2.

**Fig. 4:**
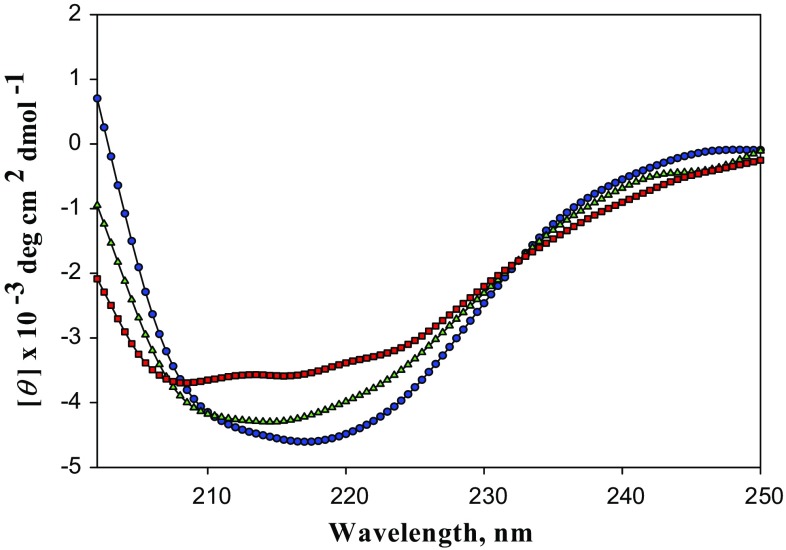
Far-UV CD spectrum of RGA2 at different temperatures in 50 mM Tris–HCl buffer pH 8.0. Far-UV CD spectrum at 25 °C, at 85 °C and at 25 °C after cooling is shown in blue, red and green, respectively

The far-UV CD spectrum provided information about secondary structure of RGA2 (Fig. 4). A deep peak at the 222 nm is indication of α-helix in the protein structure. This far-UV CD spectrum was analyzed to determine secondary structure content, we used K2D2 server (Perez-Iratxeta and Andrade-Navarro 2008). The secondary structure convoluted from the spectrum was found to be 18 % of α-helix, 32 % β-Sheet and 50 % are of random coil. We also determined α-helical content of the protein using CD value at 222 nm (Correa and Ramos 2009) to confirm our data obtained by K2D2. We used the procedure given by Morrisett et al. (1973) to determine the amount of α-helix in our protein which came out to be 16 % which is in good agreement with that obtained by K2D2. Thus these results confirmed that our protein has a significant amount of β-Sheet with α-helical content and random coil in different proportion. The crystal structure of RGA2 has not been determined so far. However, we tried to model the RGA2 using the sequence (GenBank: ACO55039.1) and observed a considerable agreement with the structure predicted through CD.

To check the stability of RGA2, we have measured the CD spectrum at 85 °C and found a significant decrease in the peak intensity at 222 nm (Fig. 4). However, it is interesting to note that this protein is not completely denatured even at 85 °C. We further checked that do such heat-induced denaturation is reversible or not? After cooling the protein from 85 to 25 °C we further measured the far-UV CD spectrum and found a significant gain in the secondary structure after cooling. These observations clearly indicate a reversible nature of RGA2 during heat-induced denaturation.

## Conclusions

Here, for the first time we are reporting a simplified procedure for the purification of RGA2 from green stems of *Tinospora cordifolia*. We have fractionated proteins of RGA2 of green stems of plants using ammonium sulfate and precipitate obtained at 90 % of ammonium sulfate was applied to DEAE-Hi-Trap FF and superdex 200 columns. The purified protein was subjected to MALDI-TOF for identification. We produced a significant amount of RGA2 from *Tinospora cordifolia* by two-step purification procedure. The method developed in our laboratory results in high yield and purity of RGA2 which may be used for further structural and biochemical studies. *Tinospora cordifolia* has an importance in traditional Ayurvedic medicine used for ages in the treatment and hence, studies on RGA2 will further strengthen our understanding for the medicinal significance of this plant.

## Electronic supplementary material

Below is the link to the electronic supplementary material.

Supplementary material 1 (PDF 124 kb)
