# Supplementary material for: Purification and characterization of RGA2, a Rho2 GTPase-activating protein from Tinospora cordifolia
Source: 3 Biotech. 2016 Mar 1;6(1):85. doi: 10.1007/s13205-016-0400-3 (PMC4773375; doi:10.1007/s13205-016-0400-3)
Supplement: Supplementary file 1 — Supplementary material 1 (PDF 124 kb) [file 13205_2016_400_MOESM1_ESM.pdf]

MATRIX SCIENCE Mascot Search Results

User	:	
Email	:	
Search title	:	AIRF-MASS\180515\MSMS 3\F9
Database	:	NCBInr 29112011 (13841106 sequences; 4750259772 residues)
Taxonomy	:	Viridiplantae (Green Plants) (872612 sequences)
Timestamp	:	18 May 2015 at 07:41:24 GMT
Warning	:	A Peptide summary report will usually give a much clearer picture of MS/MS search re
Top Score	:	38 for gi|168043302, predicted protein [Physcomitrella patens subsp. patens]

Probability Based Mowse Score

Protein score is -10*Log(P), where P is the probability that the observed match is a random event.
Protein scores greater than 72 are significant (p<0.05).
Protein scores are derived from ions scores as a non-probabilistic basis for ranking protein hits.

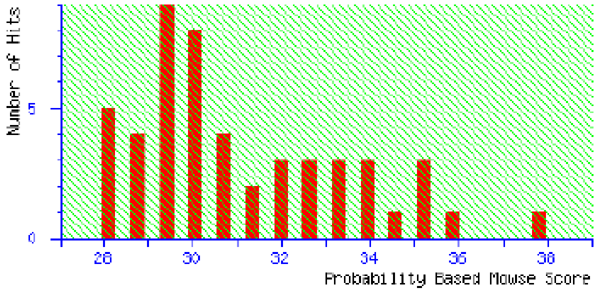

Protein Summary Report

Format As | Protein Summary (deprecated) | Help

Significance threshold p< 0.05 | Max. number of hits 20

Standard scoring (selected) | MudPIT scoring | Ions score or expect cut-off 0 | Show sub-sets 0

Show pop-ups (selected) | Suppress pop-ups | Sort unassigned Decreasing Score | Require bold red

Re-Search All | Search Unmatched

Index

	Accession	Mass	Score	Description
1.	gi|168043302	50546	38	predicted protein [Physcomitrella patens subsp. patens]
2.	gi|226427700	37906	36	RGA2 [Triticum aestivum]
3.	gi|242037791	22472	35	hypothetical protein SORBIDRAFT_01g005082 [Sorghum bicolor]
4.	gi|255553478	11489	35	conserved hypothetical protein [Ricinus communis]
5.	gi|168016420	13715	35	predicted protein [Physcomitrella patens subsp. patens]
6.	gi|3130064	40557	34	glutathione reductase [Zea mays]
7.	gi|255547732	15884	34	DNA binding protein, putative [Ricinus communis]

8.	gi|297808731	110603	34	hypothetical protein ARALYDRAFT_489530 [Arabidopsis lyrata subsp. lyr
9.	gi|242033933	20660	34	hypothetical protein SORBIDRAFT_01g016950 [Sorghum bicolor]
10.	gi|297744939	77628	33	unnamed protein product [Vitis vinifera]
11.	gi|224129126	75900	33	predicted protein [Populus trichocarpa]
12.	gi|297791899	7796	33	hypothetical protein ARALYDRAFT_917625 [Arabidopsis lyrata subsp. lyr
13.	gi|18403397	20224	33	DNA-binding protein-related [Arabidopsis thaliana]
14.	gi|307108758	49963	33	hypothetical protein CHLNCDRAFT_143635 [Chlorella variabilis]
15.	gi|168048274	28775	32	predicted protein [Physcomitrella patens subsp. patens]
16.	gi|125539201	13994	32	hypothetical protein OsI_06970 [Oryza sativa Indica Group]
17.	gi|302803985	178924	32	hypothetical protein SELMODRAFT_422911 [Selaginella moellendorffii]
18.	gi|255090068	22696	32	predicted protein [Micromonas sp. RCC299]
19.	gi|15238813	50912	31	unknown protein [Arabidopsis thaliana]
20.	gi|170517116	60875	31	maturase K [Capparis tenuisiliqua]

Results List

1. gi|168043302 Mass: 50546 Score: 38 Expect: 1.4e+002 Queries matched: 19

predicted protein [Physcomitrella patens subsp. patens]

Observed	Mr(expt)	Mr(calc)	ppm	Start		End	Miss	Ions	Peptide
1286.6412	1285.6339	1285.6878	-41.87	427	-	437	1	---	K.EPLVVEESTKR.K
1286.6412	1285.6340	1285.6878	-41.84	427	-	437	1	---	K.EPLVVEESTKR.K
1343.6670	1342.6597	1342.7344	-55.60	215	-	226	0	---	R.DPNESLLLTLTK.K
1402.7107	1401.7034	1401.6459	41.0	284	-	294	1	---	K.HTTANCWNLRK.Q + 2 Deamidated (N
2045.1415	2044.1342	2043.9189	105	243	-	258	0	---	R.HKPTNMRPNVWCSNCK.G + Oxidation
2113.1018	2112.0945	2112.0527	19.8	81	-	99	0	---	K.GNYIIFQNSTFSPASQPIK.L + Deamida
2204.2395	2203.2322	2202.9721	118	242	-	258	1	---	K.RHKPTNMRPNVWCSNCK.G + 3 Deamida
2223.2183	2222.2110	2222.2423	-14.06	24	-	43	0	---	R.ALAGNFVNIPVEILRPLTER.E + Deamid
2238.2239	2237.2166	2237.1427	33.1	349	-	369	1	---	K.GKNPIQNLDDPIEVTTVAPSK.R + 2 Dea
2301.2925	2300.2852	2299.9824	132	1	-	20	0	---	-.MCLDNGNSDDTLESYVPLEK.L + Deamid
2301.2925	2300.2852	2299.9824	132	1	-	20	0	---	-.MCLDNGNSDDTLESYVPLEK.L + Deamid
2386.2585	2385.2512	2385.2638	-5.29	206	-	226	1	---	K.ELTNELVSRDPNESLLLTLTK.K + Deami
2415.3357	2414.3284	2414.1934	55.9	131	-	155	1	---	R.GTKNSMAAGGPAVPIAIQMGTLGDR.T + 2
2608.5586	2607.5513	2607.2453	117	44	-	68	0	---	R.EGGIEVFGTPLDNFPIGSTAETSNR.G
2682.4673	2681.4600	2681.2313	85.3	1	-	23	1	---	-.MCLDNGNSDDTLESYVPLEKLPR.A + Oxi
2684.4211	2683.4138	2683.1993	80.0	1	-	23	1	---	-.MCLDNGNSDDTLESYVPLEKLPR.A + 2 D
2880.6545	2879.6472	2879.5604	30.2	372	-	397	1	---	R.TEPISVLGRILILSLQQDEEPTSVSR.A
2880.6545	2879.6473	2879.5604	30.2	372	-	397	1	---	R.TEPISVLGRILILSLQQDEEPTSVSR.A
3449.6487	3448.6414	3448.7052	-18.48	69	-	99	1	---	R.GSTDWHELVANKGNYIIFQNSTFSPASQPIK

No match to: 964.5239, 974.4750, 1020.5522, 1020.5522, 1068.5607, 1391.7384, 1416.6941, 1419.7502, 1419.7502, 1618.9102, 1646.9170, 1748.9526, 1963.0583, 1963.0584, 2019.0903, 2061.0452, 2062.1135, 2063.0630, 2063.0630, 2076.1387, 2077.1108, 2098.0520, 2110.1155, 2110.1155, 2120.0564, 2295.2498, 2335.2795, 2335.2795, 2372.2488, 2372.2488, 2392.2910, 2398.3918, 2449.3235, 2449.3235, 2556.2993, 2578.5425, 2594.5779, 2595.4866, 2595.4866, 2625.4780, 2625.4780, 2652.4731, 2822.6741, 2822.6741, 2838.9402, 2852.6738, 2852.6738, 2863.6985, 2879.7070, 2893.7249, 2909.6633, 2910.6426, 2910.6426, 3347.7407, 3361.7214, 3377.7063, 3377.7063, 3391.6934, 3395.6047, 3409.6343, 3787.0146, 3803.0164, 3803.0164, 3805.9165

2. gi|226427700 Mass: 37906 Score: 36 Expect: 2.3e+002 Queries matched: 13

RGA2 [Triticum aestivum]

Observed	Mr(expt)	Mr(calc)	ppm	Start		End	Miss	Ions	Peptide
974.4750	973.4677	973.5080	-41.38	178	-	185	0	---	R.QLDTLDIR.G + Deamidated (NQ)
1402.7107	1401.7034	1401.7517	-34.45	1	-	12	1	---	-.RWIAEGYPGVVR.N
1416.6941	1415.6868	1415.7442	-40.55	293	-	305	1	---	K.LGVMGINEKNDVK.F
